# Frequent gross deletions in *pol* gene in 10 HIV-1 infected patients treated with Korean red ginseng for 3 years: dosage dependency

**DOI:** 10.1186/1742-4690-10-S1-P15

**Published:** 2013-09-19

**Authors:** Young-Keol Cho, Jung-Eun Kim, Ba-Reum Kim

**Affiliations:** 1 Microbiology, University of Ulsan College of Medicine, Seoul, Republic of Korea

## Background

To my knowledge, there is only one report on gross deletions in the *pol* gene (gΔpol) [1] although there are many reports on gross deletions in the *nef* gene (gΔnef). It is known that Korean red ginseng (KRG) slows depletion of CD4 T cells in human immunodeficiency virus type 1-infected patients [2]. We reported an association between KRG intake and gΔnef [3] in 10 HIV-1 infected patients treated with KRG. Here, we investigated whether KRG intake also induces gΔpol.

## Materials and methods

This study consisted of 10 patients (men;women: 8;2) infected with HIV-1 subtype B. All patients had consistently taken pure KRG powder for 42 ± 4 months (mean total KRG over this time, 4,197 ± 1,278 g) for up to 3 years, with at least five blood samples available. The average monthly dose was 99 ± 25 g (range, 51 to 146 g). The daily dose was 5.4 g for men and 2.7 g for women. They were not treated with antiretroviral therapy. We amplified the *pol* gene (1,232 bp encompassing terminal portion of reverse transcriptase and integrase region) using 68 PBMC samples from these 10 patients. The *pol* gene was amplified using two primer sets, outer primers OBP1/ OBP2 (nt. 3733 to 3752/ 5297 to 5278) and inner primers OBP3/OBP4 (nt. 3837 to 3860/ 5049 to 5068). Nucleotide positions were based on NL4-3.

## Results

We obtained 277 amplicons of the *pol* gene in the 10 patients. Among the 277 amplicons, 25 were grossly deleted. There was no amplicon with a stop codon. Size of deletion was 660 ± 277 bp (49 to 1008). Seven patients exhibited gΔpol, ranging from 4.8% to 19.2%, with significant increases after KRG intake relative to baseline (12.3% vs. 1.7%) (*p*<0.05). Interestingly, 3 of 4 patients who took KRG <100 grams per month did not reveal any gΔpol, whereas all 6 patients who took KRG > 100 grams per month revealed gΔpol (>10% of amplicons). The proportion of gΔpol increased about 2-fold over the first 6 months on KRG and was statistically significantly higher after 6 months. Median time to the first detection of gΔpol was 22 months.

## Conclusion

These findings show that occurrence of gΔpol is associated with KRG intake.
